# Development and validation of an LDCT-based deep learning radiomics nomogram for predicting postoperative recurrence of stage Ia lung adenocarcinoma

**DOI:** 10.3389/fonc.2025.1706104

**Published:** 2026-01-12

**Authors:** Haimei Lan, Chaosheng Wei, Yiming Luo, Mingzhuang Liao, Hongfeng Liang, Jianli Qin, Jixing Yi, Fengming Xu, Dandan Huang, Meiqing Zhang, Qing Feng, Tao Li

**Affiliations:** 1 Department of Radiology, Liuzhou Workers’ Hospital, Guangxi, China; 2 Department of Radiation Therapy, Guangxi Medical University Cancer Hospital, Guangxi, China

**Keywords:** deep leraning radiomics nomogram, low-dose computed tomography, lung adenocarcinoma, predictive model, ResNet50

## Abstract

**Objective:**

This study wanted to use low-dose computed tomography (LDCT) plain scan images to create a deep learning radiomic nomogram (DLRN) to accurately predict the likelihood of recurrence after surgery in patients with stage Ia lung adenocarcinoma (LUAD).

**Methods:**

We collected cases from January 2010 to December 2020 at Center 1 who underwent surgery and were pathologically diagnosed with stage Ia LUAD, and additionally collected patients with the same criteria at Center 2 from January 2015 to December 2018 for external validation. Deep learning and radiomic feature extraction were performed on LDCT images of all patients. In the deep learning and radiomics methods, we tested multiple different models and selected the best model based on the results of the internal validation cohort. Finally, we construct a nomogram by combining deep learning features, radiomics features and clinical data. Subsequently, We used the receiver operating characteristic (ROC) curve to check how well these models performed in terms of diagnosis. The calibration degree of each model was evaluated using calibration curves, while the clinical value of each model was assessed through decision curve analysis (DCA).

**Results:**

In Center 1, we collected a total of 233 eligible patients, who were randomly divided into a training cohort (163 patients) and an internal validation cohort (70 patients) at a 7:3 ratio. And we collected included a total of 89 patients in Center 2. Internal validation results showed Resnet50 and Logistic Regression (LR) as optimal models for deep learning and radiomics approaches, respectively. The area under the curve (AUC) values for this combined model were 0.972 (95% CI: 0.949-0.995) in the training cohort, 0.925 (95% CI: 0.845-1.000) in the internal validation cohort, and 0.915 (95% CI: 0.853-0.976) in the external validation cohort. Compared with other single models, it demonstrated the best performance.

**Conclusion:**

Preoperative DLRN based on LDCT plain scan images exhibit good predictive value for postoperative recurrence in patients with stage Ia LUAD. The present study developed a novel prognostic assessment method with the objective of assisting clinicians in refining adjuvant treatment plans for patients with stage Ia LUAD, thus facilitating personalised prognostic management.

## 1 Introduction

Lung cancer remains the leading cause of cancer death globally (1). Non-small cell lung cancer (NSCLC) accounts for approximately 80% of all lung cancer cases, and lung adenocarcinoma (LUAD) is the most common histological type (2, 3). Recently, the widespread adoption of low-dose computed tomography (LDCT) screening for lung cancer has led to the detection of numerous early-stage LUAD patients (4–6). For these patients, surgical resection is the preferred option for achieving radical treatment (3, 7). However, postoperative recurrence is the most common cause of death and a major factor affecting long-term survival after surgery. Studies have found that the postoperative recurrence rate in patients with stage Ia LUAD is approximately 10%-20% (8). Therefore, assessing the recurrence of stage Ia LUAD after surgery is crucial for formulating personalized and effective treatment strategies.

Radiomics is a practical quantitative medical imaging technique that analyses high-throughput features derived from manually delineated tumour regions (9, 10). This technology has been extensively studied in post-operative recurrence prediction and survival analysis for LUAD (11). Traditional manual radiomics approaches merely extract surface image features from annotated regions, failing to fully capture tumour heterogeneity and thus limiting the field’s potential (12). The autonomous extraction of quantitative surface features from medical images using deep learning techniques represents a novel direction in radiomics development. Convolutional neural networks (CNNs)-based deep learning has become a promising approach for predicting postoperative recurrence in lung adenocarcinoma (LUAD), demonstrating significant clinical value (13). However, the effective application of deep learning requires substantial training data support, and medical datasets often suffer from data scarcity due to their limited scale.

In recent years, deep transfer learning (DTL) technology has emerged as a focal point of scholarly research. Its core principle lies in accomplishing novel tasks by fine-tuning pre-trained datasets, thereby enabling the application of deep learning techniques even with limited datasets. Concurrently, radiomics and deep learning have become rapidly advancing frontier technologies (14). Numerous researchers employ a transfer learning (TL) approach involving pre-trained CNNs to address overfitting issues arising from limited datasets (15, 16). Furthermore, the integration of DTL classification networks with traditional manual radiomics frameworks has gained traction in medical research (17, 18). Nevertheless, its application in postoperative recurrence studies of stage Ia LUAD remains relatively constrained, whereas the predominant research focus has been on its role in therapeutic decision-making for mid-advanced stage lung cancer (19–22).

Despite the growing body of research literature on early-stage LUAD, there remains a lack of studies employing radiomics and deep learning techniques utilising LDCT images to predict postoperative recurrence in stage Ia LUAD patients. Moreover, LDCT represents the most promising imaging modality for early screening of LUAD, effectively reducing mortality rates among lung cancer patients. This study aims to develop and validate a deep learning radiomic (DLR) feature based on LDCT images, and to explore its predictive efficacy for postoperative recurrence in stage Ia LUAD patients.

## 2 Materials and methods

**Figure 1:**
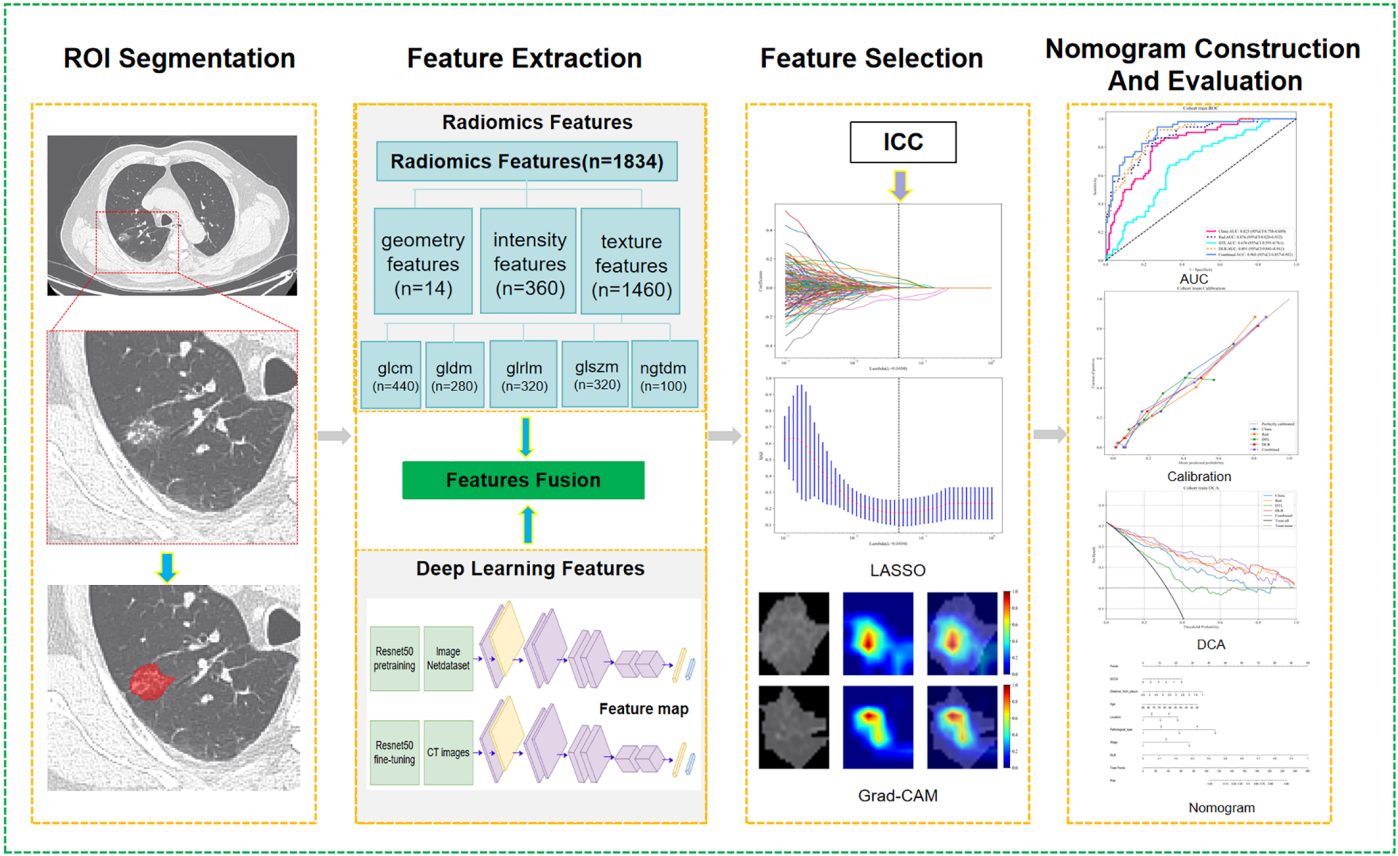
The workflow of LDCT-based DLRN.

This study introduces a DLR model using LDCT to predict the likelihood of recurrence after stage Ia LUAD surgery. Initially, handcrafted features were extracted from CT images using the pyradiomics packet. Secondly, deep learning features are extracted through the maximum cross-section of the region of interest (ROI). These features are further enhanced using TL techniques, leveraging a pre-trained ResNet50 model. A radiomics signature and a corresponding nomogram were then developed and validated on an independent cohort. **Figure 1** depicts the workflow of the radiomics analysis conducted in this research.

### 2.1 Study population and follow-up

**Figure 2:**
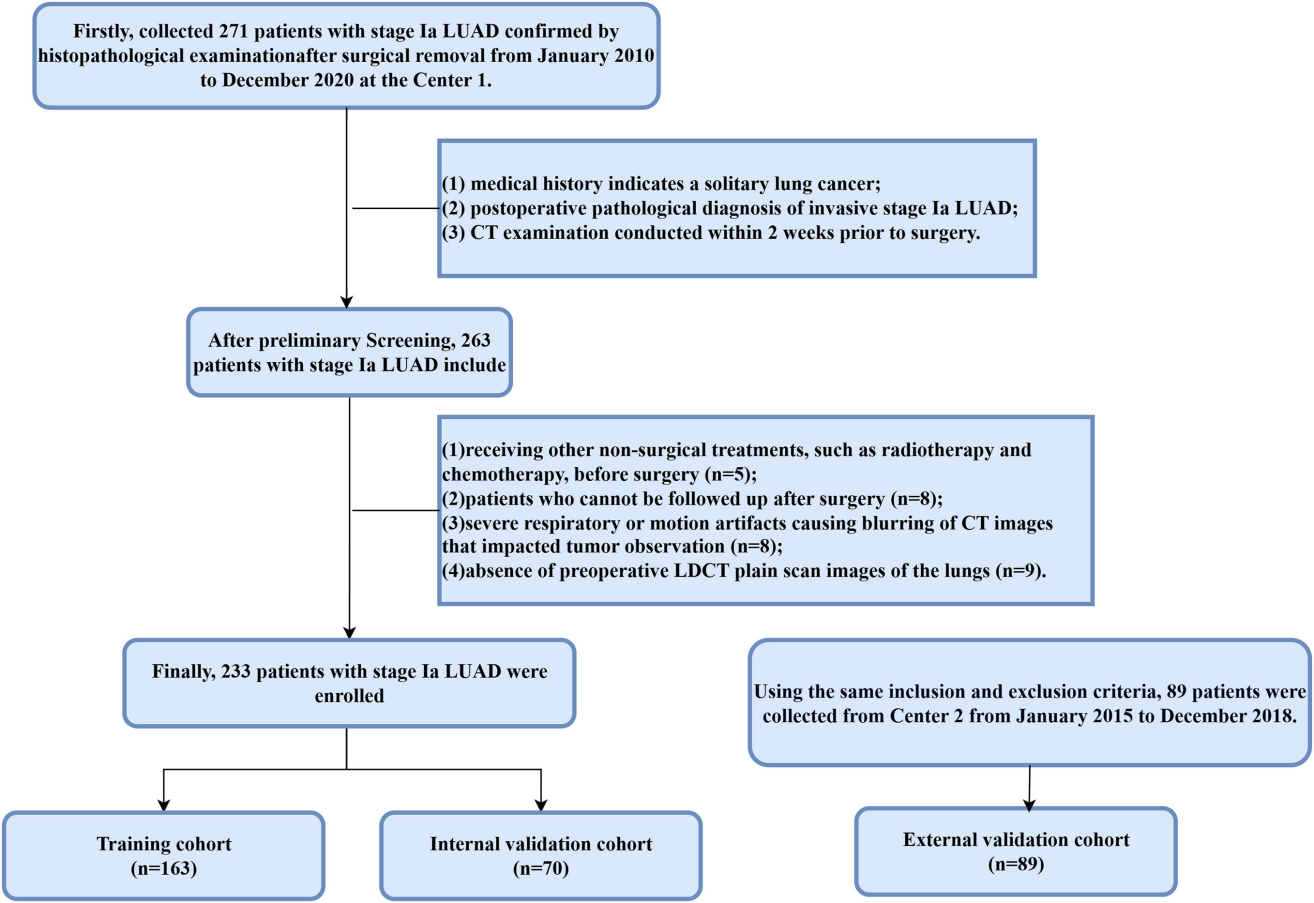
The patient recruitment process and distribution in the training and validation cohorts.

This retrospective study received approval from two institutional review boards (Approval No.: KY2025613, KY20251082) and was exempted from the requirement for patient informed consent. Data were collected on cases of surgically resected, pathologically confirmed stage Ia LUAD from Center 1 (January 2010 to December 2020) and from Center 2 (January 2015 to December 2018). Inclusion criteria: (1) medical history indicates a solitary lung cancer; (2) postoperative pathological diagnosis of invasive stage Ia LUAD; (3) CT examination conducted within 2 weeks prior to surgery. Exclusion criteria: (1) receiving other non-surgical treatments, such as radiotherapy and chemotherapy, before surgery; (2) patients who cannot be followed up after surgery; (3) severe respiratory or motion artifacts causing blurring of CT images that impacted tumor observation; (4) absence of preoperative LDCT plain scan images of the lungs. **Figure 2** illustrates the detailed recruitment methodology.

The main objective of this study is to evaluate the recurrence-free survival (RFS). Patients were followed up for 5 years after surgery. In these five years, if the tumor recurred, they were assigned to the recurrence group; If there is no recurrence, it is classified as a non-recurrence group. Follow-up once every six months for the first two years and once a year thereafter. Follow-up includes CT, MRI or PET/CT scanning, as well as telephone consultation. According to the standard research procedure, recurrence can be divided into local recurrence and distant metastasis. If the tumor reappears in N1 or N2 lymph nodes, mediastinum, primary lung position or pleura, it is considered as local recurrence. Distant metastasis refers to the spread of cancer to adrenal gland, kidney, bone, brain, liver, contralateral lung, skin or N3 lymph nodes (23).

### 2.2 CT image acquisition

All CT examinations were conducted during a breath-hold at deep inspiration, with the patient’s arms raised above their head, and the acquisition spanned from the lung apices to the lung bases. In Center 1, the scanning machine was SIEMENS SOMATOM Definition Flash (Stellar). And in Center 2, the scanning machine was General Electric Optima CT670. The CT scanning parameters for both machines were as follows: tube voltage at 120 kV, tube current at 30 mA, collimated detector at 128×0.6 mm, matrix at 512×512, and slice thickness at 5 mm. The images were reconstructed using iterative reconstruction techniques, and the acquired CT data were saved in the DICOM format. The CT images were preprocessed through standardization, including grayscale value discretization and image resampling, followed by Gaussian filtering.

### 2.3 ROI acquisition

In our study, two experienced radiologists delineated the ROI along the tumor edges on the training dataset using ITK-SNAP, while avoiding the pleural wall, large bronchi, and blood vessels during the delineation process. Any inconsistencies they found while drawing the contours were adjusted for by a doctor with more than 20 years of experience in radiology diagnosis.

### 2.4 Intraclass correlation coefficient

We used intraclass correlation coefficient (ICC) to assess radiologists ‘consistency in drawing ROI. A random sample of 100 patients was selected from the training cohort, with two physicians independently performing ROI delineation. An ICC value exceeding 0.75 was interpreted as indicating high consistency of results.

### 2.5 Radiomics procedure

Handcrafted features fall into three distinct groups: geometric, intensity-based, and textural characteristics. Researchers typically derive these features through established techniques like the Gray-Level Co-occurrence Matrix (GLCM), Gray-Level Run-Length Matrix (GLRLM), Gray-Level Size Zone Matrix (GLSZM), and Neighboring Gray-Tone Difference Matrix (NGTDM). For this particular investigation, we extracted a comprehensive set of 1,834 handcrafted features, which broke down to 14 geometric attributes, 360 intensity-related measurements, and a substantial 1,460 textural descriptors. All features were obtained using the internal procedures of Pyradiomics (http://pyradiomics.readthedocs.io).

The extracted features were standardized using Z-score normalization to ensure comparability. We then performed t-tests to assess statistical significance, keeping only those features with p-values under the 0.05 threshold. To address potential multicollinearity issues, we calculated Pearson’s correlation coefficients between feature pairs and eliminated any with correlations surpassing 0.9. The feature selection process was finalized through the least absolute shrinkage and selection operator (LASSO) regression with 10-fold cross-validation, which helped determine the ideal regularization parameter (λ). This approach produced a streamlined collection of highly predictive and meaningful features.

Machine learning algorithms, including Logistic Regression (LR), Support Vector Machine(SVM) and so on for the radiomics risk model construction. Comparative analyses were conducted to assess the performance of each model.

### 2.6 Deep learning procedure

We used the maximum cross-section of the ROI for each case as the representative image. To simplify the algorithmic analysis and minimize background noise, we retained only the smallest bounding rectangle encompassing the ROI, expanded by an additional 10 pixels based on recent research emphasizing the significance of peritumoral regions.

We performed Z-score normalization on the images input into the model to unify the intensity distribution across the RGB channels. During the training process, a real-time data augmentation strategy was also employed, including methods such as random cropping, horizontal flipping, and vertical flipping. For validation images, we limited processing to normalization.

**Figure 3:**
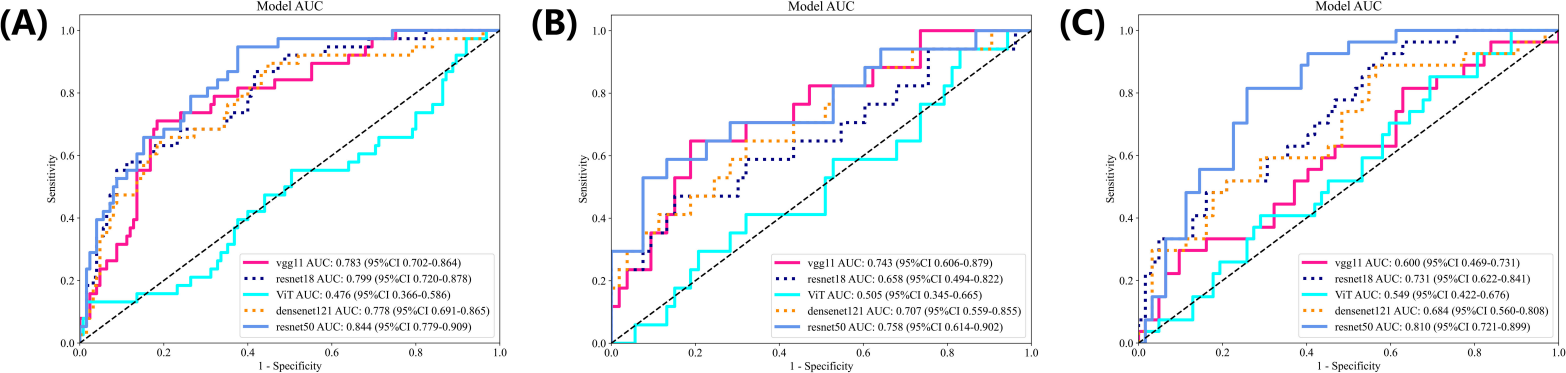
The ROC curves for different deep learning models in training **(A)**, internal validation **(B)** and external **(C)** cohorts.

During the research process, we explored the performance of the classic VGG11, ResNet18, VIT, DenseNet121 and ResNet50 networks. Moreover, an examination of these models pinpointed ResNet50 as the superior algorithm most aligned with the research goals (**Figure 3**).

In the study, transfer learning was employed to ensure the model’s effectiveness in patients with significant differences. This approach starts by loading pre-trained ImageNet weights to improve the model’s adaptability to various datasets. An important component of our approach is the continuous integration of learning rates to optimize generalization performance. To achieve this, we utilized the cosine decay learning rate strategy.


$$\eta_{t}=\eta_{\min}^{i}+\frac{1}{2}(\eta_{\max}^{i}-\eta_{\min}^{i})(1+cos(\frac{T_{cur}}{T_{i}}\pi ))$$

The minimum learning rate is initialized as $\eta_{min}^{i}=0$, whereas the upper bound is fixed at $\eta_{max}^{i}=0.01$. Here, $T_i=30$ represents the total epoch count during iterative model training. Other key hyperparameters include Stochastic Gradient Descent (SGD) for optimization and softmax cross-entropy serving as the objective function.

Feature Early Fusion: We integrated $features{ct}$, $features{dwi}$ using a feature concatenation method ($\oplus$), combining these into a single comprehensive feature vector:


$$feature_{fusion}=features_{ct}⊕features_{dwi}$$

We got 2048-dimensional depth features from the trained ResNet50 model, and also extracted 1834-dimensional standard image features from the lesion area-these features include shape, first-order features and texture features. In order to make the CNN model simpler, we PCA-processed the 2048-dimensional depth features output from the penultimate layer and compressed them into 512 dimensions. Then, we standardized the 512-dimensional depth features and the 1834-dimensional image omics features with the Z-score method. Finally, the processed feature vectors are put together to form a unified 2346-dimensional fusion feature vector, which is directly sent to the downstream logistic regression classifier for training and testing. We choose this early fusion method in order to make the classifier use the complementary information contained in the depth feature and the artificial design feature at the same time. These preprocessing steps, including Principal Component Analysis (PCA) and standardization, are critical to achieve balanced and effective fusion.

PCA can turn the initial variables into a set of independent principal components by finding the vertical axis that best represents the data changes. This method is reliable, because it can reduce the reconstruction error while retaining the most information, and also reduce the influence of feature repetition and noise. In order to test whether the features we selected are reliable or not, we used bootstrap validation, and made a total of 500 repeated sampling. In each test, we will write down which features are selected, and then calculate a score called Jaccard stability index, and the result is 0.78. We also found that eight features were particularly stable and were selected in more than 80% of the tests. Our findings also show that when we use LASSO method to select features and control the adjustment parameter λ within the range of [0.01, 0.1], the selected feature combinations are almost the same every time. However, if the wrapper-based method is used, the effect is particularly vulnerable to the parameters set at the beginning. We also draw a stability-performance trade-off curve, and find that if the selection standard is set at 0.75, we can get the most stable feature combination, and the performance of the model will not deteriorate, because the AUC score can be kept above 0.85.

### 2.7 Signature building

In the model, the CNN’s output probabilities are referred to as the Deep Learning Signature within the model.

We construct a DLR feature signature through a pre-fusion algorithm. This approach first fuses deep learning features with radiomics features, and then completes feature selection and model establishment according to the standard procedures of traditional radiomics.

To strengthen the practical utility of our findings, we performed both univariable and stepwise multivariable analyses across clinical variables to pinpoint statistically significant predictors. These key features were then merged with the predictions generated by our DLR model to construct a LR linear model—ultimately yielding what we term the Combined Signature. For enhanced clinical interpretation, this composite score was presented visually through an easy-to-use nomogram.

### 2.8 Statistical analysis

All data analyses were performed using Python 3.7.12 on the OnekeyAI platform (version 4.9.1). For clinical characteristics, continuous variables are expressed as mean ± standard deviation, while count variables are presented as percentages. **Table 1** shows the clinical baseline characteristics. Independent risk factors were identified through univariate and multivariate analyses. The DLR model utilized LASSO regression analysis and ROC analysis. Both the clinical model and the DLR model determined the optimal model based on the ROC curve. The calibration accuracy of the models was assessed using calibration curves, and the clinical utility was evaluated using DCA curves. The DeLong test was employed to assess the differences among multiple models. All reported p-values are from two-tailed tests, with a p-value less than 0.05 considered statistically significant.

**Table 1:** Baseline characters of our cohorts.

Clinical and CT features	Training cohort (n=163)	Internal validation cohort (n=70)	*P* value	External validation cohort (n=89)	*P* value
Age	59.95 ± 9.45	60.40 ± 8.70	0.734	58.06 ± 8.69	0.113
WBC	6.83 ± 1.94	6.69 ± 1.89	0.742	14.05 ± 68.12	0.321
NEU	4.17 ± 1.60	4.04 ± 1.40	0.806	4.20 ± 2.18	0.503
CEA	5.45 ± 9.57	6.37 ± 15.76	0.377	4.03 ± 3.09	0.150
CYFRA211	3.13 ± 1.87	3.10 ± 1.69	0.726	2.78 ± 1.06	0.317
NSE	13.47 ± 4.31	13.59 ± 5.80	0.433	13.09 ± 3.88	0.621
CA125	17.00 ± 62.75	14.75 ± 16.24	0.387	14.55 ± 13.96	0.037
CA153	16.02 ± 18.45	13.27 ± 6.56	0.88	15.66 ± 18.59	0.583
SCCA	1.21 ± 1.22	1.17 ± 0.82	0.596	1.19 ± 0.72	0.405
CA50	9.58 ± 11.51	18.61 ± 63.12	0.074	10.47 ± 15.99	0.712
CA242	7.59 ± 19.22	5.83 ± 7.36	0.081	7.23 ± 7.63	0.827
CA724	4.30 ± 11.24	2.97 ± 3.44	0.525	3.83 ± 6.34	0.274
Pathological type	2.56 ± 1.21	2.34 ± 1.24	0.236	2.54 ± 0.88	0.994
Distance from pleura	1.34 ± 0.88	1.36 ± 0.88	0.863	1.26 ± 0.75	0.480
Smoking			0.415		0.077
No	116(71.17%)	49(70.00%)		64(71.91%)	
Yes	47(28.83%)	21(30.00%)		25(28.09%)	
Family history			0.919		0.717
No	120(73.62%)	55(78.57%)		68(76.40%)	
Yes	43(26.38)	15(21.43%)		21(23.60%)	
Sex			0.133		1.000
Female	89(54.60%)	30(42.86%)		49(55.06%)	
Male	74(45.40%)	40(57.14%)		40(44.94%)	
Location			0.087		0.716
Left superior lobar	48(29.45%)	22(31.43%)		33(37.08%)	
Right superior lobar	45(27.61%)	30(42.86%)		20(22.47%)	
Right middeep learninge lobar	15(9.20%)	4(5.71%)		7(7.87%)	
Right inferior lobar	35(21.47%)	7(10.00%)		15(16.85%)	
Left inferior lobar	20(12.27%)	7(10.00%)		14(15.73%)	
Stage			0.508		0.965
T1a	17(10.43%)	4(5.71%)		10(11.24%)	
T1b	89(54.60%)	41(58.57%)		49(55.05%)	
T1c	57(34.97%)	25(35.72%)		30(33.71%)	
Nodule type			0.848		0.608
mGGN	66(40.49%)	30(42.86%)		32(35.96%)	
SN	97(59.51%)	40(57.14%)		57(64.04%)	

WBC, white blood cell; NEU, neutrophils; CEA, carcinoembryonic antigen; CYFRA211, cytokeratin 19 fragment assay; NSE, neuron-specific enolase assay; CA, carbohydrate antigen; mGGN, Mmixed ground-glass nodule; SN, Solid nodules.

## 3 Results

### 3.1 Clinical features

We performed detailed univariate and multivariate evaluations of clinical characteristics. These ratios were crucial in developing our final fusion model. Significant features identified through univariable and multivariate analysis screening were utilized to construct the Clinical Signature.

Univariate analysis and multivariate analysis identified sex, squamous cell carcinomaassociated antigen (SCCA), pathological type and nodule type as independent risk factors for postoperative recurrence of stage Ia LUAD (**Table 2**).

**Table 2:** Univariable and Multivariate Analysis of clinical features.

Clinical and CT features	Univariable analysis		Multivariable analysis	
	OR (95%CI)	*P* value	OR (95%CI)	*P* value
Sex	0.298(0.189-0.470)	0.000	0.179(0.047-0.683)	0.035
SCCA	0.335(0.248-0.451)	0.000	0.200(0.054-0.739)	0.043
Distance from pleura	0.508(0.409-0.629)	0.000	1.201(0.702-2.054)	0.575
Smoking	1.217(0.675-1.688)	0.618		
Family history	0.875(0.480-1.597)	0.715		
Nodule type	0.565(0.399-0.799)	0.007	12.106(2.550-57.455)	0.008
Stage	0.648(0.571-0.737)	0.000	0.579(0.232-1.448)	0.327
Pathological type	0.802(0.726-0.884)	0.000	1.9608(1.212-3.168)	0.021
CYFRA211	0.903(0.837-0.973)	0.026	1.100(0.984-1.230)	0.158
Location	0.726(0.652-0.809)	0.000	1.012(0.677-1.511)	0.962
NEU	0.792(0.738-0.849)	0.000	2.191(1.005-4.778)	0.098
WBC	0.856(0.820-0.894)	0.000	0.512(0.270-0.972)	0.086
NSE	0.920(0.899-0.942)	0.000	0.842(0.691-1.024)	0.150
CA125	1.000(0.995-1.004)	0.827		
CA242	0.921(0.884-0.959)	0.001	1.014(0.981-1.048)	0.480
Age	0.982(0.976-0.986)	0.000	0.988(0.944-1.034)	0.658
CA153	0.977(0.962-0.991)	0.010	1.017(0.993-1.041)	0.242
CEA	0.995(0.985-1.018)	0.721		
CA50	0.954(0.930-0.980)	0.004	1.030(0.972-1.092)	0.400
CA724	0.995(0.973-1.017)	0.704		

### 3.2 Radiomics signature

We assessed model discrimination across cohorts using the area under the curve AUC as the key measure. The results from the internal validation cohort indicated that LR demonstrated significantly superior performance (AUC = 0.809), outperforming SVM (AUC = 0.801) and Adaptive Boosting (AdaBoost) (AUC = 0.795). The AUC values for k-NearestNeighbor (KNN) and Extreme Gradient Boosting (XGBoost) were relatively lower and close to each other, at 0.761 and 0.772, respectively (**Table 3**).

**Table 3:** Metric results for radiomics signature.

Model	Cohort	Accuracy	AUC	95% CI	Sensitivity	Specificity	PPV	NPV
LR	Training	0.779	0.876	0.822-0.929	0.895	0.744	0.515	0.959
SVM	Training	0.828	0.886	0.833-0.939	0.921	0.800	0.583	0.971
KNN	Training	0.822	0.895	0.848-0.941	0.868	0.808	0.579	0.953
XGBoost	Training	0.933	0.970	0.944-0.996	0.921	0.936	0.814	0.975
AdaBoost	Training	0.798	0.935	0.899-0.970	1.000	0.736	0.535	1.000
LR	Internal validation	0.743	0.809	0.706-0.912	0.941	0.679	0.485	0.973
SVM	Internal validation	0.700	0.801	0.707-0.907	1.000	0.604	0.447	1.000
KNN	Internal validation	0.800	0.761	0.633-0.888	0.706	0.830	0.571	0.898
XGBoost	Internal validation	0.757	0.772	0.654-0.891	0.824	0.736	0.500	0.929
AdaBoost	Internal validation	0.686	0.795	0.716-0.915	1.000	0.585	0.436	1.000
LR	External validation	0.697	0.796	0.707-0.892	0.889	0.613	0.500	0.927
SVM	External validation	0.753	0.771	0.672-0.870	0.667	0.790	0.581	0.845
KNN	External validation	0.640	0.720	0.612-0.829	0.852	0.548	0.451	0.895
XGBoost	External validation	0.674	0.720	0.605-0.835	0.741	0.645	0.476	0.851
AdaBoost	External validation	0.685	0.723	0.612-0.834	0.704	0.677	0.487	0.840

The AUC analysis indicates that LR performs exceptionally well in both training and validation cohorts, maintaining a robust discriminative capability. While all models exhibit a decline in AUC from training to validating, LR shows the highest AUC, suggesting its generalizability and stability. The decline in AUC for all models in the validation cohorts highlights the challenge of maintaining model performance on unseen data, emphasizing the need for robust validation and potential overfitting mitigation strategies.

### 3.3 Grad-CAM visualization

**Figure 4:**
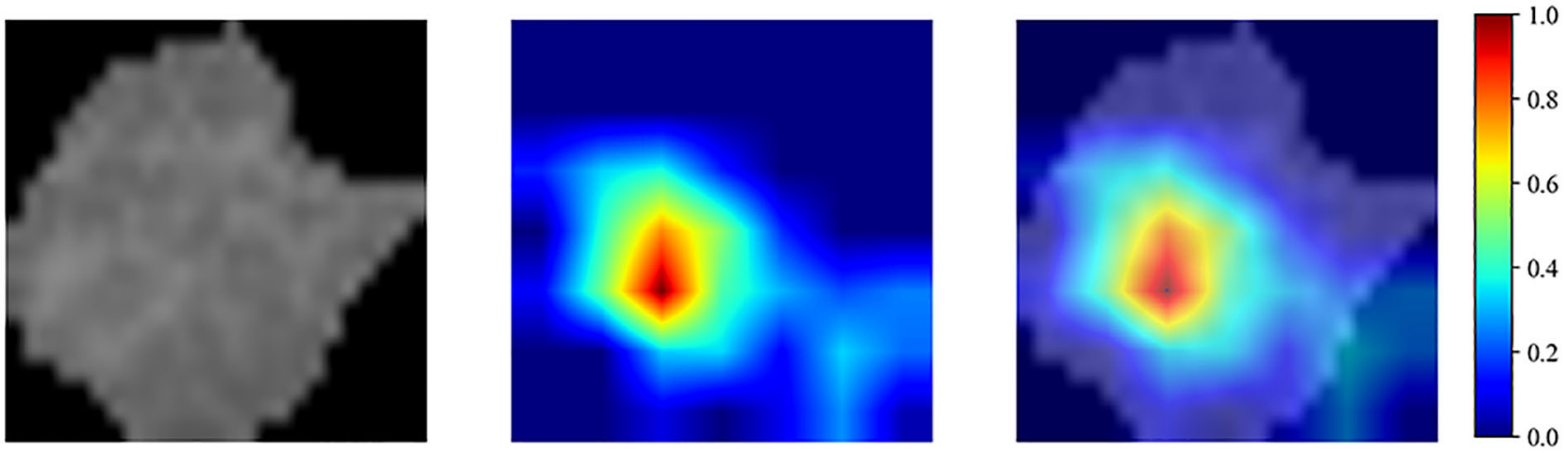
The ResNet50 model with Grad-CAM was used on stage Ia LUAD, the red area is the basis of decision-making for ResNet50.

We employed the Gradient-weighted Class Activation Mapping (Grad-CAM) technique to visualize the decision-making basis of the deep learning model, in order to investigate its recognition capability for different classes of cases. **Figure 4** presents the visualization results based on Grad-CAM, where the highlighted areas indicate the image features on which the model’s decision is based, and these features are associated with postoperative recurrence of stage Ia LUAD. By identifying image regions that are crucial for the model’s decision-making, this analysis enhances the interpretability of the model, thereby improving its reliability and clinical trustworthiness as an auxiliary tool.

### 3.4 Signature comparison

**Figure 5:**
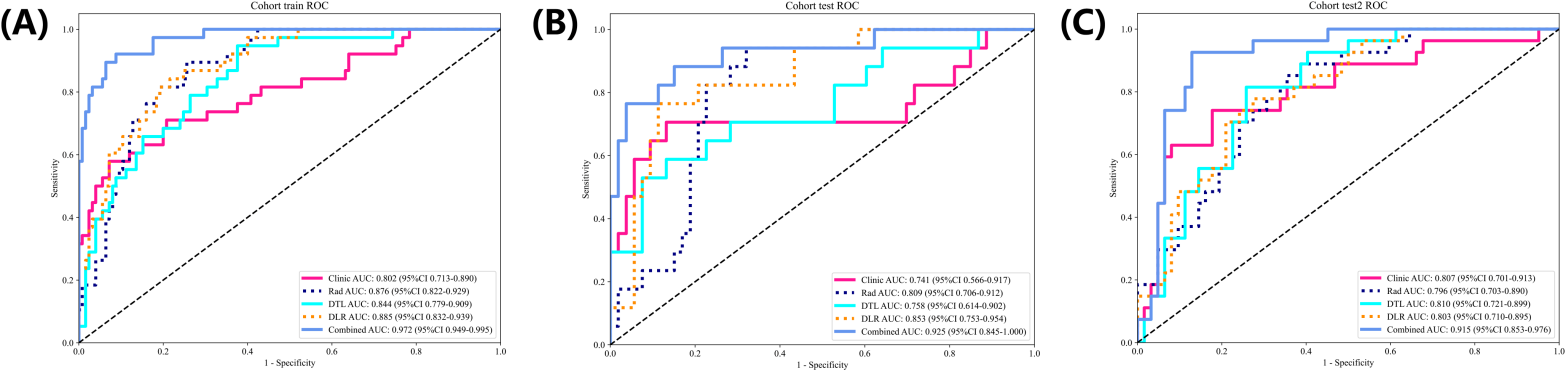
The ROC curves for different models in training **(A)**, internal validation **(B)** and external **(C)** cohorts.

The AUC values for the various signatures indicate varying levels of discriminatory power. The “Combined” signature outperforms all others, achieving the highest AUC scores in both the training (0.972) and validation (0.925, 0.915) cohorts—clear evidence of its exceptional ability to differentiate between classes. Not far behind, the “DLR” signature holds its own with impressive AUC values of 0.885 (training), 0.853 (internal validation) and 0.803 (external validation), showcasing robust classification performance. Similarly, the “Rad” signature delivers solid results, posting AUCs of 0.876, 0.809 and 0.796 in the respective cohorts. On the flip side, the “Clinic” signature lags significantly, with lackluster scores of 0.802 (training), 0.741 (internal validation) and 0.807 (external validation), highlighting its relatively weak discriminatory capacity. Meanwhile, the “DTL” signature lands squarely in the middle of the pack, with AUC values of 0.844, 0.758 and 0.810, reflecting moderate predictive performance (**Table 4** and **Figure 5**).

**Table 4:** Metrics on different signature.

Signature	Cohort	Accuracy	AUC	95% CI	Sensitivity	Specificity	PPV	NPV
Clinic	Training	0.847	0.802	0.713-0.890	0.579	0.928	0.710	0.879
Rad	Training	0.779	0.876	0.822-0.929	0.895	0.744	0.515	0.959
DTL	Training	0.699	0.844	0.779-0.909	0.947	0.624	0.434	0.975
DLR	Training	0.798	0.885	0.832-0.939	0.842	0.784	0.542	0.942
Combined	Training	0.914	0.972	0.949-0.995	0.921	0.912	0.761	0.974
Clinic	Internal validation	0.829	0.741	0.566-0.917	0.706	0.868	0.632	0.902
Rad	Internal validation	0.743	0.809	0.706-0.912	0.941	0.679	0.485	0.973
DTL	Internal validation	0.800	0.758	0.614-0.902	0.588	0.868	0.588	0.868
DLR	Internal validation	0.857	0.853	0.753-0.954	0.765	0.887	0.684	0.922
Combined	Internal validation	0.857	0.925	0.845-1.000	0.882	0.849	0.652	0.957
Clinic	External validation	0.798	0.807	0.701-0.913	0.741	0.823	0.645	0.879
Rad	External validation	0.697	0.796	0.703-0.890	0.889	0.613	0.500	0.927
DTL	External validation	0.764	0.810	0.721-0.899	0.815	0.742	0.579	0.902
DLR	External validation	0.742	0.803	0.710-0.896	0.778	0.726	0.553	0.882
Combined	External validation	0.888	0.915	0.853-0.976	0.926	0.871	0.758	0.964

Based on the AUC analysis, the “Combined” signature emerges as the most robust indicator, demonstrating high discriminatory power in both training and validation cohorts. The “DLR” and “Rad” signatures also exhibit strong performance, although slightly lower than the “Combined” signature. The “Clinic” signature, in contrast, displays the weakest discriminatory ability. These findings highlight the potential utility of the “Combined” signature in clinical or diagnostic applications, given its superior ability to differentiate between classes. These preliminary results require further validation in larger and more representative populations.

**Figure 6:**
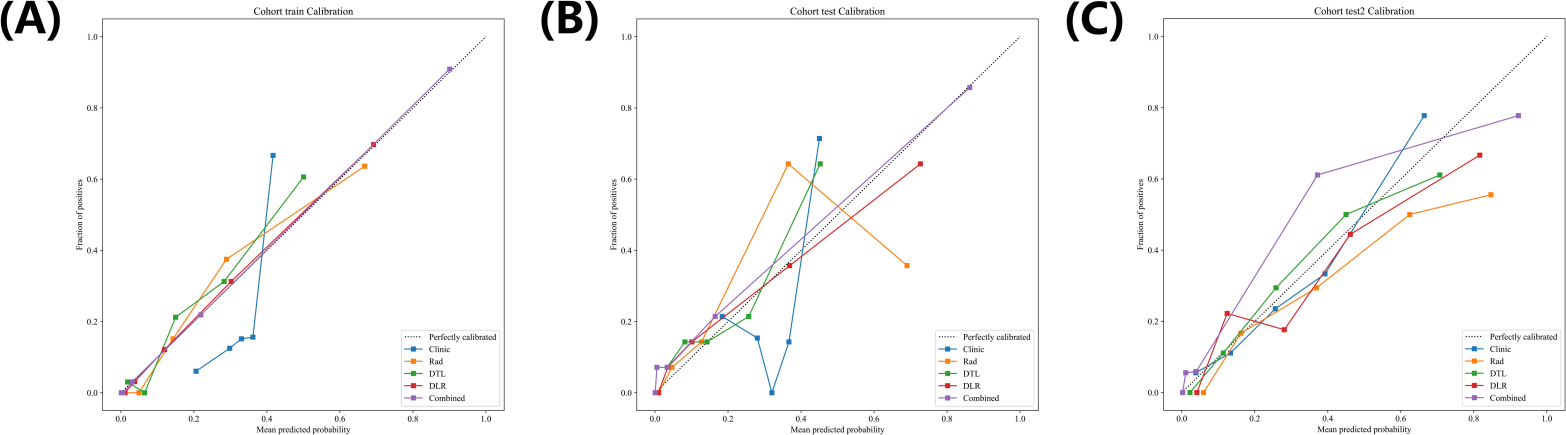
The Calibration curves of different signatures in in training **(A)** and internal validation **(B)** and external **(C)** cohorts.

**Figure 6** compares the calibration curves of each model, and the results show that the joint model has the highest calibration degree.

**Figure 7:**
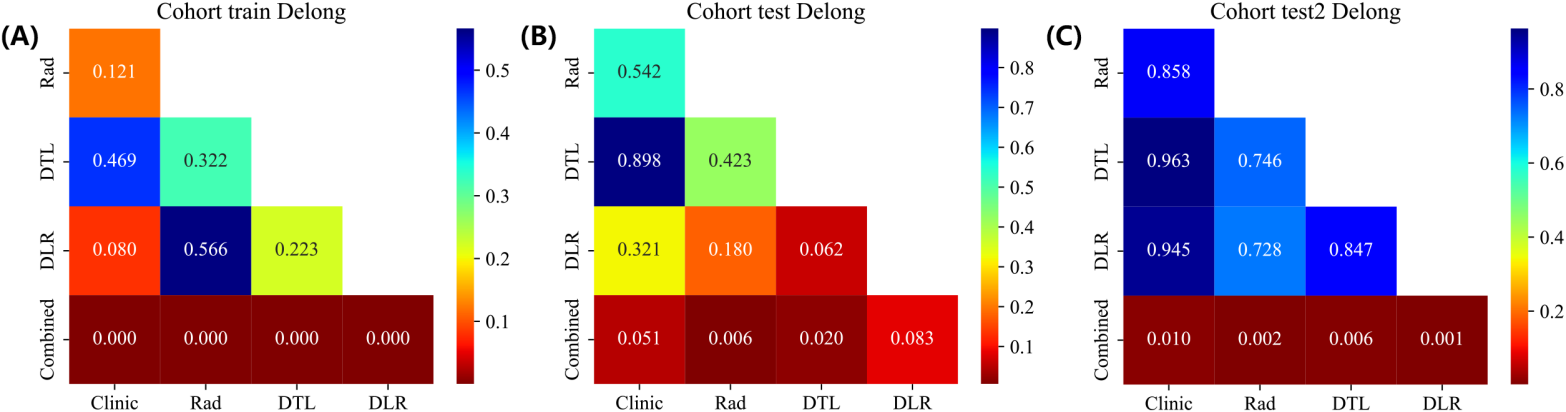
DeLong results in training **(A)** and internal validation **(B)** and external **(C)** cohorts.

To evaluate the statistical significance of performance differences, the DeLong test was applied to compare the models on the training and validation datasets (**Figure 7**). Our combined model exhibited a significant enhancement compared to other models.

### 3.5 Clinical use

**Figure 8:**
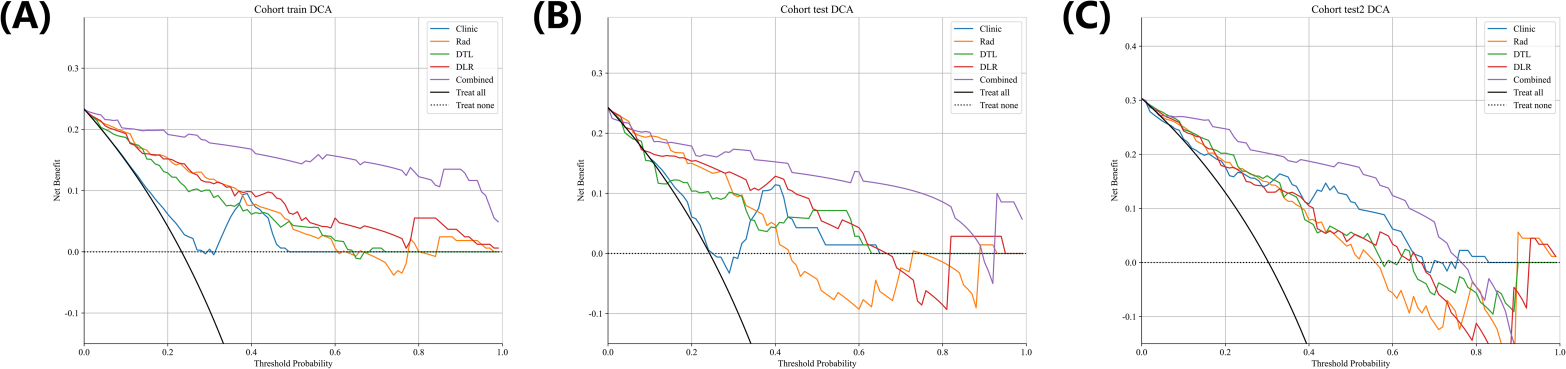
Different models’ DCA curves in training **(A)** and internal validation **(B)** and external **(C)** cohorts.

DCA: **Figure 8** illustrates the decision curve analysis for the training, internal and external validation cohorts. The findings reveal that our combined model provides considerable improvements in predicting the likelihood of recurrence in stage Ia LUAD following surgery. Moreover, it consistently outperforms other signatures by delivering a greater net benefit, underscoring its effectiveness.

### 3.6 Construction of nomogram

**Figure 9:**
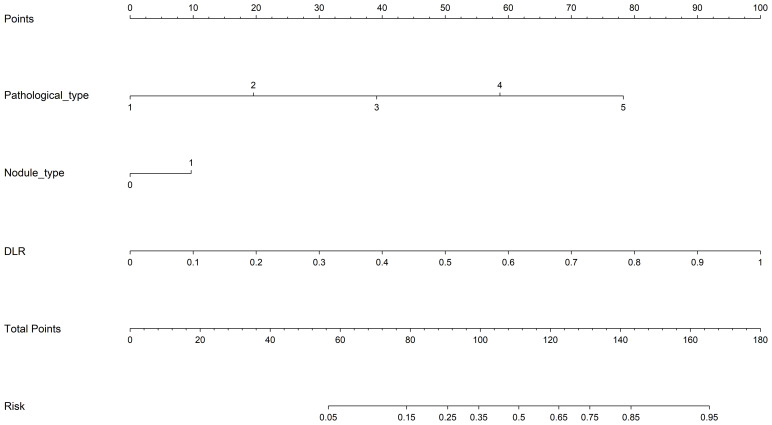
The nomogram for predicting stage Ia LUAD.

We have established a final model that integrates clinical independent risk factors with the predictive results of the DLR model and effectively visualizes them through a deep learning radiomics nomogram (DLRN). The nomogram indicates that the DLR factor plays a crucial role in the stratified prediction of postoperative recurrence risk. (**Figure 9**).

## 4 Discussion

Our study reveals that DLR features based on LDCT images exhibit distinct efficacy compared to clinical feature models in predicting postoperative recurrence, and when integrated with clinical features, they can further enhance the predictive value for postoperative recurrence in patients with stage Ia LUAD. Integrating radiomic features, deep learning features, and clinically independent risk factors into nomograms demonstrates outstanding diagnostic performance in the training internal and external validation cohorts. This fully validates the incremental value of the combined model in associating with individualized disease-free survival in patients with stage Ia LUAD after surgery.

Radiomics has emerged as a continually evolving key direction in the field of medical image analysis, providing a novel method to convert medical images into quantitative features that can reveal tumor-related biological information. By analyzing these features, clinical decision-making can be optimized (9). This technology transcends the limitations of human visual perception, achieving comprehensive representation of tumor-related information by capturing more detailed heterogeneous characteristics of tumors (24). In a study led by Su and colleagues, they utilized a fusion of clinical and radiomics techniques, anchored by CT scans, to gauge the likelihood of bone metastasis in LUAD patients. The findings showcased that this integrated approach provided exceptional diagnostic prowess (with an AUC of 0.866 and a 95% confidence interval of 0.786 to 0.947), which in turn has the potential to enhance individualized treatment planning (25). Xie et al. constructed and validated a radiomics nomogram for prognostic prediction and identification of surgical patients with stage I LUAD who may benefit from adjuvant chemotherapy (26). Zhang et al. found that radiomics has the potential ability to assess the prognosis of patients with pathological stage I LUAD (≤3cm), which may take a step forward in precision medicine (27).

In addition, an increasing number of scholars are utilizing deep learning techniques to predict the prognosis of patients undergoing surgery for LUAD, and have mentioned the practicality of this method (28–30). For instance, scholars such as Yuki et al. employed deep convolutional neural networks (DCNN) to predict postoperative recurrence of LUAD using preoperative CT images. The results confirmed that DCNN technology utilizing preoperative CT images can effectively predict postoperative recurrence in patients undergoing LUAD surgery (13). Scholars such as Zhu have confirmed that deep learning algorithms can replace manual methods for tumor measurement, and they outperform manual measurement in the prognostic stratification of patients with LUAD (31). In a study by Wang et al. employing radiomics to predict early recurrence, they extracted peritumoural deep learning features and established a radiomics model incorporating tumour spread through air spaces model. The results demonstrated its potential in guiding adjuvant postoperative treatment strategies (32). Peng and other scholars developed the ResNet50 architecture to evaluate the major pathological response (MPR) in patients with lung squamous cell carcinoma (LUSC) after receiving neoadjuvant chemoimmunotherapy (NCI) treatment. They divided the data into a discovery set (n=200), validation set 1 (n=60), and validation set 2 (n=49). Finally, it was found that the ResNet50 model trained on enhanced CT images was developed and validated for predicting MPR, and its AUC in the first and second validation sets was 0.95 and 0.90, respectively (33).

An increasing number of studies are integrating DTL classification networks with conventional artificial radiomics features (34, 35). However, the use of DLR features based on LDCT images to assess postoperative recurrence in patients with stage Ia LUAD has not been identified. In this study, by utilizing the deep learning-radiomics-clinical combined model we proposed, we obtained results superior to those previously reported. This result indicates that the risk of postoperative recurrence in surgical patients with stage Ia LUAD can be predicted using preoperative LDCT images through a combined model. Our study only requires LDCT images, which we believe have a unique advantage, which they enable individualized postoperative recurrence assessment under preoperative noninvasive conditions.

Univariate and multivariate regression analyses revealed that sex, SCCA levels, pathological type, and nodule type are independent predictors of postoperative recurrence in patients with stage Ia LUAD, which can be used to establish a clinical model. Miyoshi and other scholars retrospectively analyzed the clinicopathological characteristics of 809 patients with stage Ia LUAD and found that patients with ground-glass opacity (GGO) nodules had significantly higher survival rates than those without GGO components (5-year overall survival rate: 97% vs. 84%, p < 0.0001). For patients in the three different stages of T1a, T1b, and T1c, those with ground-glass nodules exhibited higher survival rates compared to those with solid nodules (36). Moreira et al. confirmed in their research that a high-grade subtype (grade 3, predominantly solid/micropapillary components) is a stronger predictor of recurrence than the TNM stage alone (37). This signifies that the pathological subtype has evolved from a qualitative description to a quantitative, standardized prognostic grading tool. This indicates that the pathological type and nodule type have a significant impact on the prognosis of lung adenocarcinoma, which has also been confirmed in this study. To date, no studies have identified sex and SCCA as independent risk factors for recurrence in LUAD. Moreover, as they were not significant in our feature assessment, they were excluded from the model construction. Therefore, we developed a nomogram that combines independent predictive factors with DLR features to predict the recurrence in patients with stage Ia LUAD. Excitingly, compared to single deep learning, radiomics, and clinical models, the nomogram demonstrates a significant improvement in the AUC. It can provide a valuable therapeutic window for patients with stage Ia LUAD who may experience recurrence, and it aids in formulating more rational and effective treatment plans.

Furthermore, the DeLong test was conducted on the AUC of each model. In the training cohort, the AUC value of the combined model showed significant differences compared to clinical model. This results indicate that the deep learning-radiomics-clinical combined model performs better than single models.

The limitations of this study are that it is a retrospective analysis, and the dataset is small. A small sample size is a prevalent and significant challenge in medical imaging research. For instance, it can lead to several adverse outcomes, including inadequate statistical power and unreliable results, distorted model performance evaluations, and the failure of internal validation. While an ideal study design would be a prospective longitudinal cohort study (38), for patients with stage Ia LUAD, conducting such a study faces significant challenges due to the long waiting period required for survival outcome data. Although the generalisability of outcomes would be more convincingly demonstrated through large-scale, independent, prospective, our DCA can assess clinical relevance, confirming that the nomogram identified holds significant potential for clinical application in predicting postoperative outcomes. Moreover, these results have also been well validated in the external validation cohort.

In summary, the identified DLR features predict postoperative recurrence in stage Ia LUAD, a finding confirmed through external validation. When compared to other clinical risk factors, the DLRN detailed herein underscores its pivotal role in the personalized assessment of postoperative recurrence. Although the conclusions of recent small-scale research are only preliminary, mainly to stimulate new ideas and explore new directions, the most critical step is to conduct comprehensive verification through large-scale and multi-center prospective experiments. Our main goal is to establish a reliable index or framework based on imaging, and test it widely in various practical environments, and finally provide a solid basis for its inclusion in clinical decision support tools in the future.

## Data Availability

The original contributions presented in the study are included in the article/supplementary material. Further inquiries can be directed to the corresponding authors.
